# Converting donor dependence to domestic ownership: The realignment of tuberculosis financing for sustainability

**DOI:** 10.1371/journal.pgph.0006523

**Published:** 2026-05-29

**Authors:** William A. Wells, Sarah Scheening, Saba Waseem, Amanda Schulhofer, Yemelaknesh Wolde, Guy Stallworthy

**Affiliations:** 1 Independent Consultant, Melbourne, Victoria, Australia; 2 Open Development, Washington, District of Columbia, United States of America; 3 Independent Consultant, Washington, District of Columbia, United States of America; 4 Independent Consultant, Alexandria, Virginia, United States of America; 5 Gates Foundation, Seattle, Washington‌‌, United States of America; Universidade Catolica Portuguesa, PORTUGAL

## Abstract

The financing of tuberculosis (TB) programs in high burden countries (HBCs) requires an overhaul. Donor funding for TB has often bypassed domestic channels, decreased the incentive for engagement between national TB programs and national budget holders, and provided minimal assistance to improve domestic TB budgeting processes. The result is local budgeting and financial management processes that do not efficiently and effectively meet both curative and public health TB needs. Meanwhile, donor TB funding has become less certain. Our analysis suggests that, in almost three quarters of high TB burden countries, increases in health budgets and in the proportion of health budgets dedicated to TB could compensate financially for a complete withdrawal of donor funding for TB. However, marginalized populations may be left behind and, in countries with less resources, major gaps will remain. Ministries of Health and Finance should assess whether the country can pay for more of its own TB response; this includes defining TB-specific financing needs, and assigning these needs to specific domestic financing sources. They should also demand that future TB donor funds flow through domestic channels, and prioritise TB in their engagement with Multilateral Development Banks (MDBs). Donors and MDBs should: renew their commitment to funding TB; ensure that TB donor funds and TB loans flow through domestic systems; communicate more clearly around transition; provide assistance related to advocacy, accountability, and the raising, allocation and more efficient use of domestic TB funding; and target TB implementation funding to lower-income countries where it is most urgently needed. The end result would be national TB programs that are system-aligned, with donor support that is more clearly differentiated between implementation and system building in lower-income countries and addressing health system constraints in middle-income countries.

## The changing context for development assistance for health

Disruptions in development assistance for health (DAH) in 2025 [1] have prompted urgent questions about global health financing. Concern about donors running “essential care programmes indefinitely in parallel” [2] and “perpetuat[ing] the need for global health institutions” [3] has led to calls for recipient countries to assert greater ownership [4]. In this context, the time is right for the topic of this article: a re-examination of the roles of domestic and donor financing for tuberculosis (TB) programs. Below, we outline the scale of TB DAH relative to other financing sources, the implications of this for the future of TB financing, and some future directions for both high TB burden countries (HBCs) and TB donors.

Since 1990, there has been seven-fold growth in DAH [5]. A greater share of that money has gone to low-middle-income countries (LMICs; seeing a 113% increase in DAH share) rather than low-income countries (LICs; seeing a 41% decrease) [5], likely due to inertia in scaling down DAH for countries as they progress from LIC to LMIC status. (Note, however, that almost three-quarters of the world’s poorest people now live in middle-income countries [6].) Finally, there has been a greater focus on four health priority areas: HIV/AIDS (in particular); reproductive, maternal, newborn, and child health (RMNCH); TB; and malaria.

Meanwhile, in LICs and LMICs, the domestic tax bases, health revenues, and budget execution rates for health have seen nowhere near such increases [7]. As a result, apart from some exceptions [8], the more prominent narratives in global health have been around donor-driven projects, partnerships and priorities, rather than domestic government institutions and budget processes.

## Changes in the current TB financing landscape‌‌

TB has been selected for support by donors for multiple reasons [9]: the externalities associated with an airborne disease that is the leading infectious disease killer globally; the economic impact (particularly on low-income communities); domestic under-spending on TB public health activities in multiple HBCs [10]; and the link to HIV.

Donor financing for TB pales in comparison to donor financing for HIV (US$ 1.2 billion vs US$ 9.7 billion in 2022), and is only 3.1% of total official development assistance (ODA) for health and population programmes (US$ 39 billion in 2022) [11]. Yet donor financing for TB is a major funding source for TB programmes. In HBCs, the proportion of external financing can be 3–30-fold higher for TB programmes than for the overall health sector [10]. In selected HBCs, donor funding represents 54% (in LMICs) to 62% (in LICs) of available TB funding [11]. It has also been overly reliant on one source: The US government provided 49% of the total amount of donor funding for TB from 2013-22 (when considering both bilateral contributions and those via the Global Fund to Fight AIDS, TB and Malaria (GFATM)), which is six times more than the next largest donor country [11]. TB funding needs may shift in coming years with introduction costs and, later, possible cost savings from new diagnostics, shorter duration therapeutics and, in the longer term, a vaccine [12], but substantial service delivery and public health costs will remain.

The past year has seen a funding crisis for the World Health Organization (WHO) [13] and more widely for global health [14], a call to focus multinationals on global public goods and fragile states with less country implementation [15], and discussions about sunsetting multiple global health initiatives [16], including the Joint United Nations Programme on HIV/AIDS (UNAIDS) [17], GAVI, the Vaccine Alliance [18], and the U.S. President’s Emergency Plan for AIDS Relief (PEPFAR) [19]. TB services have also been impacted. TB program funding through the US Agency for International Development (USAID) has been disrupted [20,21], and GFATM reduced current grants by an average of 11% [22]. This led to disrupted TB services [23,24], including in South Africa [25] and Nigeria [26]. TB transmission modeling suggests these and other potential cuts would result in 1–4 million additional TB cases and 0.5-1 million additional deaths (or even higher losses [27,28]) by 2035 [29].

## Making harder choices: Differentiating by country gross national income

How would such cuts impact different countries? We analyzed 40 HBCs [30] for their amounts of TB funding from donors, their other DAH, and their domestic health financing (i.e., general government health expenditure – domestic (GGHE-D)) [31]. We conclude that a significant share of TB donor support goes to countries that should be capable of replacing all donor TB financing with domestic financing – albeit with a risk that low-income and marginalized populations get left behind.

**Fig 1:**
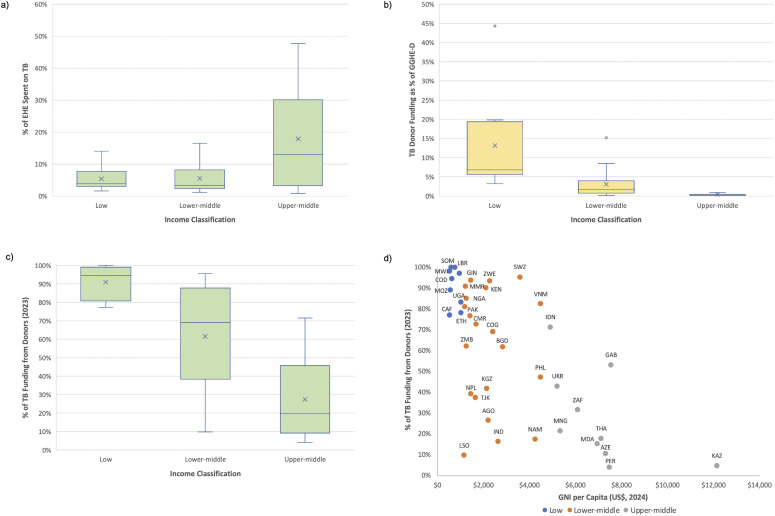
The relative scale of donor funding for TB. **(a)** The percentage of External Health Expenditure (EHE) spent on TB in each country income group. This demonstrates that TB donor funding is a small proportion of total donor funding in LICs, but a larger proportion in UMICs. Countries analysed were the 49 high TB burden countries identified by the WHO [30], minus 9 countries: Brazil, China, and Russia because they had no GFATM or USAID funding; and Belarus, Botswana, North Korea, Papua New Guinea, Sierra Leone, and Uzbekistan due to insufficient recent financing data. The TB donor funding received in 2023 was from WHO’s Global TB Report (rcvd_tot_sources) [32], defined as the sum of contributions from GFATM, USAID, and other external sources. These amounts were divided by EHE based on data from the WHO Global Health Expenditure Database for the most recent year available (2022, except Philippines and Somalia [2021], and Ukraine [2019]); the same data source was used in Fig 1b for domestic general government health expenditure (GGHE-D) data [33]. Gross national income (GNI) per capita data for the most recent year available (2024) was drawn from the World Bank [34] and each country was assigned an income level classification according to the World Bank’s 2025 GNI per capita thresholds [35]. **(b)** TB donor funding as a percentage of domestic health expenditure, by income group. TB DAH is significant compared with total domestic health funding in LICs, but small relative to domestic health funding in UMICs. The y axis illustrates the fiscal space required if countries assumed full financing of their current TB programs, which is far higher in LICs. Outliers are Somalia (LIC) and Myanmar (LMIC). **(c)** Distribution of TB donor dependency by income group. TB donor dependency was high in LICs, low in UMICs, and variable in LMICs. **(d)** TB donor dependency by GNI per capita. Countries are identified by the three-letter ISO 3166 Country Codes [36], and color-coded based on their World Bank income level. The highly variable donor dependency of LMICs is clearly visible.

The relative levels of TB DAH are depicted in Fig 1. In LICs, the amount of TB DAH is small compared to total DAH (Fig 1a) but significant compared with domestic health funding (Fig 1b). In upper middle-income countries (UMICs), the reverse is true: TB DAH is a larger proportion of total DAH (Fig 1a) but is small relative to total domestic government health expenditure (Fig 1b). Therefore, LICs will need to continue to tap DAH funds, whereas in UMICs it is feasible for domestic sources (GGHE-D) to fund the gap left by declining TB DAH.

Consistent with this, and not surprisingly, TB donor dependency (the percentage of total TB program funding coming from donors) is high in LICs and low in UMICs. This is evident in both country groupings (Fig 1c) or individual countries (Fig 1d), after plotting by Gross National Income (GNI) per capita. However, there is huge variability in TB donor dependency within LMICs. Thus, the vulnerability of countries to TB donor cuts is not predictable based solely on the level of GNI.

**Fig 2:**
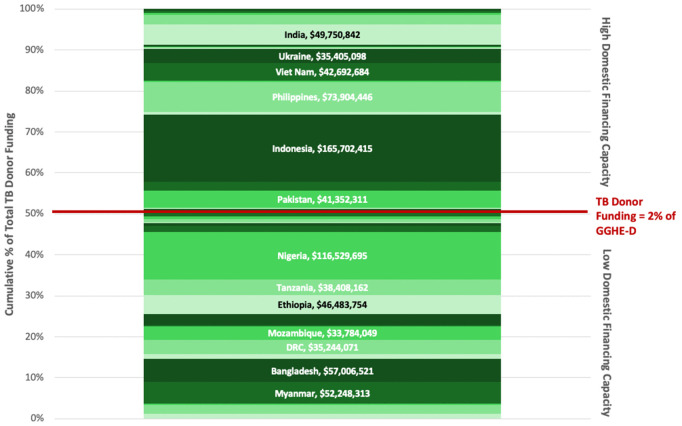
The percentage of total TB Donor Funding to 40 HBCs (2023), per country, with countries ranked from least to most able to absorb domestically. Countries at the bottom of this figure are those least able to compensate for TB donor funding declines by using GGHE-D, since they score the highest for the variable “TB DAH as % of GGHE-D”. Countries at the top are the most able to compensate for TB donor funding declines using GGHE-D, since their TB DAH is low compared to their GGHE-D. The size of the country in the graphic reflects the absolute dollar amount of TB donor funding for that country, and all countries covering >3% of TB donor funding are labeled. Based on this graphic, Myanmar, Bangladesh and Nigeria are major TB donor funding recipients with a relatively lower ability to replace that donor funding with GGHE-D, whereas Philippines, Indonesia, South Africa and India are countries with a higher ability to replace TB donor funding with GGHE-D.

A significant proportion of TB DAH goes to countries that are quite able to absorb the financial cost domestically. In Fig 2, the 40 HBCs are ordered from those least able to compensate for TB DAH declines by using GGHE-D (at the bottom) to those most able, using the variable “TB DAH as % of GGHE-D” to order the countries. The size of the country’s colored section reflects the absolute dollar amount of TB DAH for that country. Almost half of total TB DAH is provided to countries in the top of the graphic; these are countries that have significant TB funding absorption capacity, where TB DAH represents <2% of GGHE-D. This persistence of TB donor funding in MICs reflects donor policies but also epidemiology: Many large MICs still have a lot of TB due to persistent poverty [6] and because TB latency results in a slow decline of TB burdens [37].

**Fig 3:**
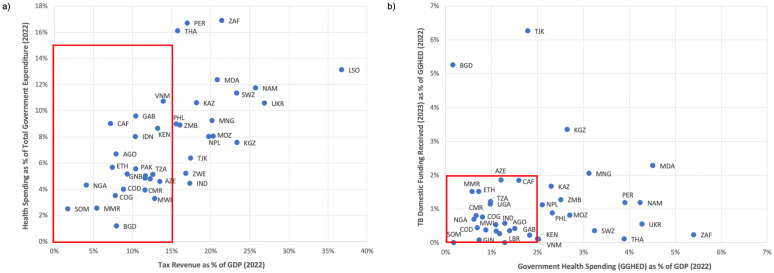
Efficacy of raising taxes and allocating government expenditure to health and TB in TB HBCs. **(a)** To visualize potential pathways to raise domestic resources for TB, we examined tax revenue as a percentage of GDP from the IMF’s World Revenue Longitudinal Database (WoRLD) [40], and GGHE-D as a percentage of general government expenditure (GGE) from the WHO Global Health Expenditure Database [33]. Countries in the lower left area of this graph are struggling both to raise sufficient tax revenue (relative to GDP) and to allocate a sufficient percentage of government expenditure to health. Those in the red box do not meet either of the 15% metrics described in the main text, whereas the three countries in the upper right corner are the only countries that are positive outliers on both of these metrics. **(b)** Countries in the lower left area of this graph are struggling both to mobilize sufficient funds for government expenditure on health, relative to GDP, and to allocate sufficient domestic health budget to TB (the red box contains countries that are below the average (for this group of countries) for the GDP metric, or below a more aspirational 2% target for the TB domestic funding metric). Those further towards the upper right are positive outliers on both of these metrics.

To what extent could broader financial reforms contribute to meeting such TB funding shortfalls? Within government budgets, an increase of domestic funds for TB can come from greater tax collection [38], greater allocation of those collected taxes to health, or greater allocation of health funds to TB. Certain HBCs are already doing well on these metrics: HBCs towards the top right of Fig 3a are collecting a lot of tax revenue relative to their GDP, and allocating a lot of government spending to health. And HBCs in the top right of Fig 3b are also allocating a lot of health money to TB. Further examination of these and other outliers may reveal lessons for other HBCs. For example, India in Fig 1d has a low GNI per capita but also low TB donor dependency, as it has used political leadership to mobilize domestic funding for TB [39].

We also projected the increase in available budget for TB if HBCs could: increase tax revenue to the “tipping point” [41] of 15% of GDP; allocate 15% of total government expenditure (GGE) to health (in line with the Abuja declaration [42]); and allocate 1.5% of health spending to TB (the mean value for the 40 HBCs). Table 1 summarizes the impact of these scenarios across the 40 HBCs. Increasing general tax revenue to 15% of GDP has the least impact. However, increasing either the health allocation or TB allocation parameters would both add around $1.5bn to aggregate spending on TB (~1.5 times DAH for TB in these countries). Implementing all three policies together would increase domestic TB funding six-fold and only eight countries, all in sub-Saharan Africa, would still need some TB DAH in order to maintain current funding levels. The continued shortfalls in eight HBCs (five LICs: Central African Republic, Guinea-Bissau, Malawi, Mozambique, Uganda; and three LMICs: Eswatini, United Republic of Tanzania, Zambia) are a particular concern. This further emphasizes that, if TB DAH continues to be limited, it will need to be more concentrated in those LICs that do not have realistic prospects of meeting their own TB funding needs, even with the most ambitious overall domestic financing realignments.

**Table 1:** Different scenarios for increasing domestic government funding for TB: impact on aggregate TB funding and donor dependence in 40 HBCs.

Scenario	Total donor TB funding US$ million	Total domestic TB funding US$ million	Amount of increase in total domestic TB funding over baseline US$ million	% increase total domestic TB funding over baseline	Increase in domestic TB funding as a percentage of total donor TB funding	Number of countries in which the increase in domestic TB funding > current donor TB funding
Baseline	1,004	970				
A: tax revenue ≥ 15% of GDP, all else same		1,130	160	16%	16%	1
B: GGHE-D ≥ 15% GGE, all else same		2,427	1,457	150%	145%	14
C: Govt TB funding ≥ 1.5% of GGHE-D, all else same		2,575	1,605	166%	160%	13
D: A + B + C		6,877	5,907	609%	588%	32

Data sources are as outlined in the legends of Figs 1 and 3.

This analysis comes with some caveats. Historically, the primary driver of increases in budgetary space for health in LMICs has been from growth in overall government expenditure rather than growth in the share of the budget allocated to health [43]. Second, funding needs will shift with technology changes. Third, TB DAH may not be a fair representation of what TB resources are needed. Fourth, the rigidity of health budgets means that simple reallocation within a health budget can be challenging (see Action 4 below). And fifth, identifying funding is just one of the many challenges of an aid transition [44–46].

Nevertheless, there are three main implications. First, there is scope for many TB HBCs to substantially increase total funding for TB through domestic government resources, even in the face of substantial reductions in DAH. Second, donors should protect and even increase TB DAH in countries with limited capacity for domestic resource mobilization in order to support service delivery as well as basic system improvements. Third, in HBCs with greater capacity for domestic financing, limited amounts of DAH should focus on advocacy [47,48] (including for marginalized populations), and technical assistance for system reforms (including the government’s ability to contract key population-led organizations) that would better align TB financing, governance and service configurations to a domestically funded response. The rest of this paper outlines practical steps that donors and HBC governments can take to increase domestic funding for TB.

## What can countries do in response?

For countries looking to fill TB gaps left by donors, there are several priority actions. Similar processes may be applicable to other disease programs that are also seeing decreasing donor support.

### 1. Determine TB-specific funding needs

TB funding needs depend in part on the relationship of the TB program to the rest of the health system. Some have argued for a dissolution of vertical disease programs [2], since the existence of multiple programs can lead to system inefficiencies [49]. Indeed, TB service delivery has long benefited from being embedded within a strong primary health care (PHC) system [50], and there is scope for further integration-related and technical efficiencies.

However, a quality TB program goes far beyond integrated curative care: To deliver on its public health mandate, a TB program requires extensive public health actions on aspects such as prevention, active case finding, private provider engagement, and adherence monitoring [10]. These critical TB public health actions, and the quality of TB care, tend to be lost without aspects of a TB-specific program, including performance targets [10]. A strong TB response does not require either a standalone TB program or an integrated approach; it requires both.

Each country must therefore assess opportunities for system alignment [51], and determine what works “well enough” with integrated financing and an integrated workforce [52], versus needing a TB-specific push (e.g., for screening of specific populations [53]). The resultant combination of vertical and integrated programs creates challenges with quantifying “TB financing”. For example, social health insurance payments and TB subnational funding are often left out of official TB funding accounts. Nevertheless, the need for TB-specific work and TB-specific financing persists, even in high income settings [54]. Determining the nature of these TB-specific financing needs is an essential input for discussions with financing stakeholders.

### 2. Convening TB and financing discussions

The TB Financial Sustainability Index (TB FSI) is a tool that countries can use to measure and plan their progress in achieving sustainable financing for TB [55–57]. Implementation of the TB FSI identified a number of issues requiring country-level action (Table 2). It also revealed an important block to past progress: an orientation of National TB Programs (NTPs) more towards donor financing processes than domestic financing systems and dialogues [57] (an understandable and predictable consequence of concentrated donor funding [58]). Historically, this led to slow growth of domestic TB financing, as noted during the TB FSI [57]. To correct this situation, convenings are needed to increase the understanding between TB and financing stakeholders [9], to advocate for TB, and to implement the actions outlined below.

**Table 2:** Findings and priorities from implementation of the TB FSI.

Findings/ challenges	Responses/ priorities for the future
NTPs are relatively siloed from financing colleagues, and there is a greater focus on donor processes than domestic financing systems	Develop domestically adapted TB financing tools (see below). Build health financing capacity within NTPs, and establish an ongoing dialogue with the Ministry of Finance (MoF) and broader financing community Avoid missed opportunities; NTPs should take advantage of broader financing reforms when they happen Strengthen domestic and regional TB advocates
TB National Strategic Plans (NSPs) are not aligned with domestic budgets: • TB needs are not assigned to domestic budget holders; • NSPs use idealized costing that is disconnected from domestic TB budgets and budget holders; • Challenges with TB resource tracking and with domestic line-item budgets, so NTPs can’t articulate what incremental funds would “buy” in terms of impact, and therefore there are few “asks”	Assign funding responsibilities. Use domestically tailored tools to crosswalk intervention budgets from different budget holders, link all budgets to outcomes, and implement an intervention-level funding gap analysis
More meetings than financial commitments in TB multisectoral accountability frameworks (MAFs)	To increase multisectoral financing for TB, link MAFs to planning and budgeting dialogues
Subnational governments are rarely accountable for how they spend their own or national TB funding	Develop a national-subnational collaboration framework: this can encompass co-financing arrangements, earmarks or incentives
Little results-based domestic financing for TB, and few countries use contracting. Variable results on the efficiency of domestic financing of TB commodities.	Build expertise in TB purchasing (both public health contracting and insurance-based service delivery); advocate for open, competitive tenders for TB commodities
“Free TB care” is policy, not reality, due to pre-diagnostic and private sector costs, and limited coverage under insurance and social protection schemes.	NTP engagement on health policy and social protection dialogues

### 3. Analyze and cross-walk existing TB budgets

Raising funds for TB is not just about budget advocacy for health [59] and TB [9]; it also requires careful alignment of funding sources with programmatic needs. There are multiple potential sources for domestic health funding (Table 3; Box 1) [60], and the nature of funding sources (e.g., their size, availability, and relative proximity to implementers) must match the specific needs of each part of the TB program [10,61]. A large proportion of TB donor funds support public health activities, so determining a domestic financing source for those activities is particularly important.

**Table 3:** Possible funding approaches for TB.

Possible funding sources for TB	Examples and experiences with TB
National budgeting	India has had significant success in mobilizing domestic resources for TB at the national level [62]. This required a sustained advocacy effort at all levels, high level leadership, and coordination with states. National frameworks guided the utilization of resulting funds, but with space for local innovation and customization. Other HBCs have responded to donor funding shortfalls, although some promised increases may be short-term bridging allocations and not institutionalized in multi-year budgets. Ethiopia increased the earmarked TB program budget by 100% this fiscal year, and Uganda nearly doubled the national health sector allocation [63].
Subnational budgeting [64]	Negotiating subnational funding for TB is more feasible when there are fewer subnational units (as in regions in Ethiopia [65]) but requires deliberate capacity building to strengthen subnational planning and budgeting [66]. Subnational funding can be particularly well suited to the funding of local public health actions, but the provision of guidance from national level is often necessary to ensure funds are allocated to TB and there is coherence in the overall program. Similarly, any effort to fund drug procurement from the subnational level should ideally be accompanied by a national-level procurement framework, which allows for efficiencies based on higher volumes. The amount of the program that can be funded subnationally depends in part on the proportion of the overall government budget at the subnational level, which will be different in every country.
Social health insurance (SHI) [67,68]	SHI is a promising avenue to support curative payments for TB to both public and private providers [69], but is rarely used to support TB public health activities [10], and SHI in most LICs and LMICs has limited population coverage and rarely uses strategic purchasing for TB [57]. TB commodities including drugs can be covered financially by either SHI or a national program. If the national program procures commodities, the country will need a mechanism to deliver these publicly funded TB drugs to SHI-empaneled private providers.
Health taxes [70]	Health taxes have attracted considerable support [71]. There are obvious links between tobacco taxes and TB, which can be used to promote TB-specific earmarks [72,73]. For example, Tanzania is increasing excise duties on alcoholic beverages and levies on fuel, minerals, betting, casinos, and travel tickets, with 70% of this additional revenue allocated to the AIDS Trust Fund, and 30% to support the Universal Health Fund [74]. However, new health taxes and levies can be regressive [75], volatile [76], and fungible (the resulting revenue may be offset by cuts from other sources) [77], with weak linkages to TB-specific spending.
Blended financing, definition #1: melding grants with loans from multilateral development banks (MDBs) to prioritize specific interventions [78]	The World Bank has made some TB-specific loans (to India for $400m [79], and Indonesia for $300m [80]) with interest rate buy-downs from GFATM. This required substantial resources from GFATM: $40m for India and $21m for Indonesia. TB funds can also be part of health system strengthening loans (though the TB objectives need to be clear so they are not lost; see Box 1). E.g., in Lao PDR, the GFATM grant was put into a joint investment with the World Bank, GAVI, the Vaccine Alliance and the Australian government to integrate TB into primary health care [81]. Loans can help protect TB allocations during the transition to domestic funding for TB, increase the focus of senior government officials on program performance, and facilitate major system reforms. There is clearly a need for more such operations, both by the World Bank and by other MDBs. Constraints include competition from other priorities (such as infrastructure investments) and concerns about country debt burden [82–84]. See also Box 1.
Blended financing, definition #2: using public or donor funds to de-risk investments and thus attract private capital [85]	For the crowding-in of private investment, there are some limited examples with TB in India [86] and Nigeria (a US$50 million public–private partnership agreement to mobilize private capital and expertise for TB service delivery [87]), though even in these cases substantial donor funding was needed to make any private investment sufficiently attractive. Across the development landscape more broadly, blended financing that attracts private capital has remained at a small scale: “a cottage industry with largely bespoke and fragmented interventions” [88]. Within TB, new product development can allow for a return on private investment, and private providers are a valuable service delivery channel for TB. But the financing of that TB service delivery should be public, since TB preferentially targets low-income populations. Relying on out-of-pocket payments to fund TB services is problematic given that almost half of TB clients already incur catastrophic costs [89]. Two blended financing variants are social impact bonds (SIBs, in which private investors get a return on their investment from the public sector if and only if a project is successful) and their close relatives, development impact bonds (DIBs, in which the payment to investors for results comes from donors). The promise of these instruments is to trigger innovation and a focus on results rather than process. However, impact bonds have suffered from multiple challenges including difficulties with the attribution of results, the possibility of adverse selection of “easier” clients, and large transaction costs and administrative burdens [90].
Earmarked debt swaps (redirecting a country’s debt payments into funding for domestic health programs)	Debt swaps have been pursued by GFATM [91,92], including for TB in Indonesia, where the German government agreed to convert €75 million of Indonesia’s debt into investments for public health including TB [93]. Recently, trilateral or commercial debt swaps have generated larger amounts of longer term funding but have not yet been used for health [94].

**Box 1. Identifying sources – including loans – for increased TB funding**

Mainstreaming of some TB functions (e.g., clinical training) into broader frameworks can reduce the amount of TB-specific financing needed. But if the planning process (Box 2) still identifies significant TB funding gaps, what are some possible pathways to fill such gaps? Out of the options outlined in Table 3, SHI is an exciting opportunity for the future but currently lacks scale in most HBCs, debt swaps are relatively rare opportunities, and leveraging of private sector financing is promising for developing new technologies but usually a poor fit for supporting ongoing implementation costs in a public health area such as TB. Subnational financing can be promising for public health activities but only if there is a national PFM and TB performance framework to make it cohesive and non-optional. Then, for the remaining gaps, the question is whether a compelling case can be made for increased national budgets for TB (particularly when TB must compete with other, often larger health programs that are also seeing decreasing donor funding), and whether the current PFM approach in the country can secure such program-specific funds.

If the national health budget is locked almost entirely in general categories such as human resources and facility costs, two possible ways to unlock TB-specific domestic money are via health taxes (with the caveats listed in Table 3) and disease-specific loans from multilateral development banks (MDBs).

Establishing such MDB loans requires political commitment, since there are debt concerns, and other sectors and other health areas are competing for a limited pool of both concessional and non-concessional loan resources. TB may struggle to win this race, since it is a disease preferentially affecting the poor and disenfranchised, and it relies extensively on public health approaches, which are politically less visible than curative care. Therefore, buydowns of TB loans by donors (in which donor funds are used to reduce or eliminate interest payments on the loan) may be an important tool to make such loans more politically attractive domestically, while also making the donor funding more catalytic. MDBs can also be more proactive in highlighting the importance of the TB funding transition in their negotiations with client governments: there have been recent calls to renew the 2005–15 International Development Association (IDA)* Malaria Booster Program (see references 95 and 96), and a similar initiative is merited for TB.

TB objectives can be easily lost in a general health sector loan, and a purely TB-specific loan may be a difficult ask. But a loan that includes TB, HIV and malaria (in African nations) or TB and non-communicable diseases (in Asian nations) may be a helpful compromise. Whatever the scope, such loans should have carefully designed disbursement-linked indicators (DLIs), as it is not easy to craft DLIs that are challenging yet achievable, and that reflect impact as well as process. These DLIs can then be used across both the loan and any related grants, as a kind of informal blended financing. Coordination between countries, donors and MDBs on strategy will be critical.

Both domestic and international stakeholders should be very clear and consistent on whether the primary objective of such a loan is to fill temporary fiscal gaps or to drive domestic reform. Time-bound reforms should be included in the loan framework and aim to avoid any possibility that the loan will establish another form of dependency. Such reforms are typically very technocratic, so there is an acute need for associated trust funds or other donors to pay for related technical assistance. Implementation of MDB loans is often facilitated by individual technical assistance providers, but some reforms may call for larger, more structured health systems reform teams that in the past were more typically financed by USAID. Such teams should focus on a small number of reforms, but in a way that cuts across all relevant health system building blocks (i.e., service delivery, health information systems, workforce, financing, access to medicines, and governance).

* The IDA is part of the World Bank group. Of note, its highly concessional loans are only available to countries with a GNI per capita below an established threshold ($1,325 in fiscal year 2026, updated annually). If applied to TB, most of the middle-income HBCs would not be eligible. They would, however, be eligible for loans from the International Bank for Reconstruction and Development (IBRD), the lending arm of the World Bank Group. IBRD terms, though concessional, are closer to market rates.

**Fig 4:**
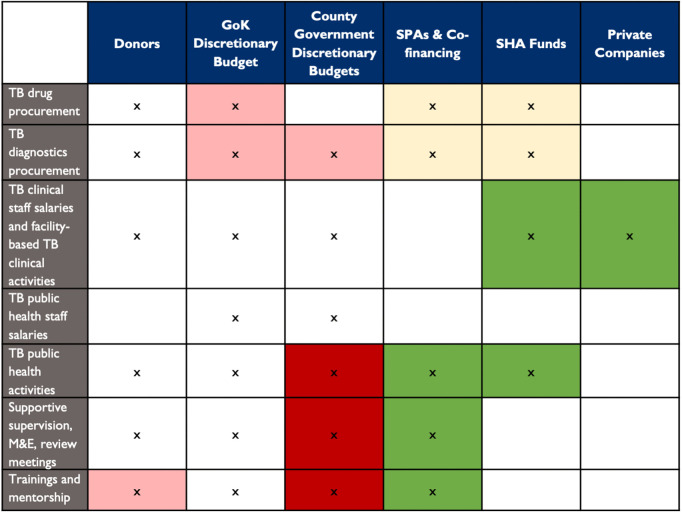
Kenya’s TB investment plan: distribution of TB contributions across funding sources and cost categories [61]. In this figure, stakeholders in Kenya projected which budget categories were expected to get less or more from each TB funding source in the future. The baseline distribution of TB contributions is captured in the white cells. The “x”s denote current or future contributions. The green cells denote cases in which there was judged to be scope to mobilize new funding for the given TB cost category by FY2027/28 (dark green) or by FY2032/33 (light green). The red cells denote cases where contributions exist at baseline but are expected to become insignificant by FY2027/28 (dark red) or by FY2032/33 (light red). Yellow indicates an area where a choice between two future funding options is needed. GOK: Government of Kenya; SPAs: Special Purpose Accounts; SHA: Social Health Authority.

One practical exercise is to develop a simple matrix to match TB budget categories to current and future TB funding sources. The general process is described in Box 2, and the results of one such matching exercise (in Kenya) is depicted in Fig 4. This process should be conducted using domestically tailored tools, since the various funding sources and their relative importance will be different in every country.

**Box 2. Planning the future state of TB financing**

Planning for improved TB financing involves the following steps:

a) Divide the TB budget into a short and practical list of broad financial categories (such as TB drug commodities, TB diagnostic commodities, TB clinical salaries, TB public health staff salaries, TB public health activities, TB supervision plus monitoring and evaluation, and TB trainings). The categories should reflect how domestic budgets are organized.

b) Crosswalk existing TB budgets from different funding sources (e.g., national budget, subnational budget, social health insurance, donors, and private sector) into the harmonized categories.

c) Use the harmonized, compiled budgets to conduct an intervention-level funding gap analysis. Use costing that is aligned with the cost structures used in domestic TB budgets and by domestic implementers.

d) Convene a TB and financing discussion to determine which of these current budget categories is expected to get less or more from each TB funding source in the future. Consider whether the proposed funding sources are a good logistical match for the corresponding budget categories within the TB program, as outlined in Table 3.

### 4. Undertake public financial management (PFM) reforms

Countries moving from a dedicated, TB-only donor fund to a more complex web of domestic financing for TB face multiple public financial management (PFM) challenges [57]. Even after matching TB budget categories to domestic TB funding sources (whether national budget, a new tax, a loan [95,96], or elsewhere; see Box 1), most countries lack the PFM architecture to link new revenue to the intended TB spending. Even new taxes generally flow into the national revenue authority, and can be allocated outside of the intended purpose unless there is a legal earmark, budget codes, and tracking systems for accountability, including at the subnational level [77].

In the section above, allocations to TB were changed via an edit to a spreadsheet. But in the real world, almost all of a health budget is already committed to existing staff and facilities, and flexible, program-based funding is rare, particularly in lower-income countries [9,97]. Though uneven in their implementation, LMICs have been moving more recently towards program-based budgeting (PBB) [98]. PBB can not only allow the identification of funding for TB, but also provide a framework for improved program performance in an integrated health financing environment [99]. Ministry of Health leadership will be needed to design and test such models.

## What is the role of donors?

Past donor efforts in sustainability and transition (summarized elsewhere [46]) have fallen short. While donors must remain committed to funding TB (to address a global public health threat that affects all countries), the global TB community must reconsider how it operates. This is based on the simple premise that countries, not donors, are ultimately accountable for achieving TB results.

To build more sustainable TB programs, a number of changes are needed in donor practices:

- Donors should place greater emphasis on clear and credible transition planning, setting out conditions and timelines for the gradual withdrawal of external funding. Transparent transition plans give countries the foresight they need to lead sustainability efforts, align financing with their own priorities, and enact reforms that strengthen domestic ownership.
- Donors should shift their TB implementation funding to LICs that have few other options. The reduced MIC funding should focus not on implementation but on catalytic technical assistance and knowledge exchange, particularly on TB-related health system constraints, including advocacy, accountability, domestic financing and PFM.
- Donors should reinforce marginal aid concepts [100] – i.e., the core of the TB program should be under domestic financing, with donor financing supporting more peripheral concerns. This contrasts with the current emphasis on “lifesaving care” [101], which usually results in continued donor investment in core program components such as commodities and front-line staff, thus consolidating donors in an ongoing role, and blocking HBCs from taking financial responsibility for their own programs.
- Donors should: avoid building duplicate systems; be clear about what they fund and how it aligns with national priorities; support programming with or advocacy by marginalized populations that may otherwise be left behind by government; and use unit costs that are known to countries.
- Donors should mainstream donor funding through national systems and, in doing so, increase their risk tolerance. It has been noted [102] that the underperformance of national PFM systems has led donors to avoid using these domestic systems – thus further weakening them. Reliance on large, stand-alone project-management units (PMUs) has further distanced both donor and loan financing from domestic systems. Flowing more TB financing through domestic systems might create short-term challenges and inefficiencies, but it is also needed to generate solutions to those challenges, and is the only way to build a sustainable and resilient system. On-budget grants [103] (and loans [104,105]) can reset expectations about how large of a domestic TB budget is required in the future.
- A continuing challenge to financial reforms in TB programs is the focus of most donor-financed TB technical assistance on the medical and TB technical aspects of TB programs, rather than on the more operational details of how such efforts are funded, staffed, and operationalized through domestic systems. As a result, significant gaps in TB financial technical assistance remain [57], including in: methods for linking TB to national planning and budgeting processes; strategic purchasing via both contracting and insurance; and measurement and planning related to TB sustainable financing.

## Conclusion

TB requires dedicated financing, and sustainable TB programs require reliable domestic sources for that financing. Our simple calculations show that the work of covering financing for TB domestically is achievable in a number of the larger HBCs. However, these HBCs will need a concerted effort to define their TB-specific financing needs, convene TB financing dialogues, crosswalk existing budgets, align funding needs and sources, and undertake PFM reforms to ensure TB financing can be assigned and tracked. In addition, TB donor support is still needed for both transition in MICs and ongoing support in lower-income countries. Significant progress will require clearer role definition and communication from donors and a rebalance in the relative effort expended on integrating TB into domestic systems compared to discussions on the medical aspects of TB programs. Medical and TB technical quality and innovations will never cease to be a priority, but the mechanics of how such efforts are implemented deserves much greater attention.
